# Supplementary material for: Are carbon emissions trading and green financial instruments synergistic? -Comprehensive quantitative research based on content analysis
Source: PLoS One. 2024 Mar 7;19(3):e0298601. doi: 10.1371/journal.pone.0298601 (PMC10919601; doi:10.1371/journal.pone.0298601)
Supplement: S1 Data — (ZIP) [file pone.0298601.s001.zip › Data/地级市Z_F工作报告.zip!/┴╔─■/╔≥╤⌠╩╨/└┤╫╘╣1⁄2╓┌║┼í╛┬φ┐╦╩2╛▌═°í┐_2014.docx]

各位代表：

　　现在，我代表市政府向大会报告工作，请予审议，并请市政协各位委员提出意见和建议。

　　一、2013年工作回顾

　　过去一年，在市委的正确领导下，在市人大及其常委会依法监督和市政协民主监督下，市政府团结带领全市人民，全面贯彻落实党的十八大精神，扎实推进“稳增长、办全运、惠民生”等重点工作，圆满完成了市十五届人大一次会议确定的各项任务。

　　——经济综合实力持续攀升。预计全市地区生产总值实现7223.7亿元，比上年增长10%；公共财政预算收入完成801亿元，增长12%；固定资产投资完成6470亿元，增长15%；社会消费品零售总额实现3222.2亿元，增长15%。

　　——产业升级步伐不断加快。规模以上装备制造业产值占工业比重由上年的47.1%提高到50.9%；高新技术产品增加值占工业比重由43.4%提高到44.2%；现代服务业增加值占服务业比重由53.1%上升到53.9%。

　　——城乡环境面貌明显改观。一批重点基础设施和配套设施项目相继竣工，市容环境治理和生态环境建设取得新成效，全市单位地区生产总值能耗下降3.77%，化学需氧量、二氧化硫排放总量分别下降2.5%和1.5%。

　　——全运筹办任务圆满完成。全面完成沈阳赛区场馆建设、赛事组织、大型活动、运行保障、安全保卫等工作，充分展示了沈阳良好的城市风貌。

　　——人民生活水平稳步提高。教育、社保、医疗等民生支出实现489.7亿元，占公共财政预算支出的55.5%。城市居民人均可支配收入实现29340元，增长11%；农民人均纯收入实现14610元，增长12%。

　　一年来，我们主要抓了以下几方面工作。

　　（一）全力以赴稳增长，经济保持平稳健康发展

　　坚持稳中求进，强化经济运行组织调度，推进重大项目加快建设，深入开展“双进双解”活动，促进了经济稳定增长和质量效益提升。

　　工业继续保持较快增长。深入实施工业“五项工程”，加快装备制造业聚集区和配套区建设，规模以上工业总产值实现13750亿元，增长14%。汽车及零部件、机械装备、现代建筑等优势产业支撑作用明显，米其林轮胎新工厂、五洲龙新能源汽车等项目投产，华晨宝马新工厂二期、上通北盛三期等项目试生产，华晨宝马发动机、沃得农机产业园等项目加快建设，全市汽车整车产量突破100万辆，手机产量突破3000万部。沈阳汽车城、铁西滨河新城、于洪永安新城等重点园区建设加快，美国安博、澳大利亚嘉民、新加坡普洛斯、意大利维龙等世界工业地产5强企业落户沈阳，25个重点产业集群销售收入实现7500亿元，沈飞、沈鼓等重点骨干企业保持较快增长，一批重大装备国产化实现新突破。

　　服务业发展水平稳步提高。服务业增加值实现3129.7亿元，增长8%。香格里拉酒店、君悦酒店、奥体万达广场、亿丰不夜城等项目竣工营业，乐天世界、华强商业金融中心、宝能环球金融中心等项目加快建设，金廊中央商务区功能进一步提升。浑南国家电子商务示范基地建设成效显著，中兴大厦、商业城等大型商业企业和五爱市场、二手车市场等专业市场积极开展网上营销，京东商城、苏宁易购等全国10强电商企业入驻沈阳。加快传统商业街区改造升级，鼓励企业开展延时服务，夜间经济更加活跃。加快东北区域金融中心建设，新增浙商银行等35家金融和投融资机构，辽宁股权交易中心正式运营。物流、会展、文化、旅游产业加快发展，房地产市场保持平稳健康发展。

　　农业和农村经济实力不断增强。强化农业基础地位，粮食总产量实现82.1亿斤，创历史新高。加快发展现代农业，全面推进蒲河生态经济带、沈康现代农业示范带、环城都市农业产业带建设，全市新开工规模以上农事项目117个，新建改造设施农业9.5万亩，高效特色农业稳定在300万亩以上，农业机械化综合水平提高到75.5%。县域工业不断壮大，法库辉山乳业婴幼儿配方奶粉加工项目建成投产，新民玖龙纸业项目加快建设，新增辽中有色金属、新民灯饰灯具2个百亿产业集群。县城和中心镇建设步伐加快，新型城镇化稳步推进。

　　新兴产业加快发展。IC装备产业位列全国三甲，新松机器人企业规模进一步扩大，东软、先锋、天久、易迅、晨讯等5家企业跻身中国软件业百强，全市软件服务业收入实现1300亿元。国家大学科技城、泗水科技城、金属新材料产业园等重点园区建设步伐加快。高档数控机床、重型燃气轮机等重大专项实现新突破，搭载I5系统的ETC数控车床实现量产，首台高性能12英寸PECVD设备达到国际先进水平。

　　（二）加大城乡建设管理力度，城市功能环境得到提升

　　以筹办全运会为契机，加大城乡建设管理力度，城市功能品位进一步提升。全年城建投资完成400亿元。

　　基础设施建设迈上新台阶。四环快速路全线通车，南二环快速路、三环高速路、伯官桥、北海高架桥等路桥建设改造竣工通车，整修261条主次干道，维修改造60条小街小巷，新建改造维修农村公路929.9公里。地铁9号线、10号线开工建设，浑南现代有轨电车试运营，桃仙机场T3航站楼、沈阳北站南广场、沈阳站东广场等重点工程投入使用。黄河大街北出口排水系统、三好排水泵站及配套工程基本完成，供热管网互联工程启动实施，康平燃气工程竣工送气，城市运行保障能力进一步增强。

　　生态环境持续改善。大力实施“青山工程”，全面开展城乡绿化，棋盘山、卧龙湖生态保护利用稳步推进。大力实施“碧水工程”，辽河、浑河、蒲河等重点流域治理扎实推进，实施封井压采，地下水资源保护得到加强。大力实施“蓝天工程”，制定出台重污染天气应急预案，电厂脱硝改造、企业“煤改气”、机动车尾气和扬尘治理等专项工程积极推进。南部污水处理厂通水运行，城市污水处理能力达到100%。生态市建设扎实推进，沈阳经济技术开发区成为东北首个国家生态工业示范园区，法库经济开发区成为国家循环化改造示范试点园区。

　　城市管理水平不断提升。开展“清洁沈阳”活动，加大拆违治乱力度，加强农村环境连片整治，城乡人居环境进一步改善。加强交通组织管理，新建97处公共停车场和10处行人过街设施，新增更新公交车1000辆，更新出租车3000辆，新开调整公交线路20条，沈阳获批国家“公交都市”创建城市。推进“数字沈阳”建设，规范设置公共信息标志，城市管理的科学化、信息化水平进一步提升。

　　（三）全面推进改革开放，经济社会发展活力不断增强

　　以综合配套改革为统领，着力推进改革开放，进一步激发了老工业基地振兴的内在动力和发展活力。

　　重点领域改革不断深化。国家新型工业化综合配套改革迈出坚实步伐，沈阳经济区优化金融生态改革试验专项方案获得国家批准，我市以第一名的成绩通过国家级两化融合试验区验收，大东区获批省级综合配套改革试验区，于洪区成为年度全国十大改革区（县）。机床、东药、沈鼓、北方重工等重点国有企业改革扎实推进，大型骨干企业分离生产性服务业取得积极进展。实行国有企业负责人经营业绩考核新办法，国资监管体制进一步完善。支持企业上市融资，沈机股份完成定向增发，辉山乳业在香港联交所上市。鼓励支持非公有制经济发展，加快中小企业服务平台建设，非公有制经济占全市经济比重为65.8%。土地经营权确权登记等农村综合改革继续深化。改革财政资金投入方式，整合完善公共资源交易中心，大力推进向市场、向社会、向下级政府放权，发展软环境进一步优化。

　　对外开放水平不断提高。全市实际利用外资58.2亿美元，实际到位内资1757亿元，进出口总额实现141.1亿美元，新签超千万美元境外工程项目35个。沈阳综合保税区加快建设，营口港法库陆港启动运营，辉山经济开发区晋升为国家级开发区。开通沈阳至新加坡等6条国际及地区航线，沈阳成为72小时过境免签城市，与俄罗斯新西伯利亚市、韩国大田市结为友好城市。

　　沈阳经济区一体化建设步伐加快。实施东陵区（浑南新区）与棋盘山旅游开发区合并，为加快建设沈抚新城和加强棋盘山生态保护提供了有利条件。加快城际连接带建设，中心城市聚集辐射能力进一步增强。

　　（四）认真做好“十二运”筹备工作，沈阳赛区服务保障任务圆满完成

　　全面完成沈阳赛区各项承办任务，为办成一届文明节俭、热烈精彩、开创新风的体育盛会作出了积极贡献。

　　筹办工作和赛事组织扎实有序。高水平推进浑南新城和全运村建设，高质量完成25个市属场馆和17个市、区两级全民健身中心建设改造。圆满完成火炬传递、开闭幕式及沈阳赛区各项赛事的组织保障工作，实现了“零事故、零差错、零失误”目标。全运期间，共接待国内外嘉宾万余人，为运动员、教练员、技术官员、媒体记者等宾客提供参观游览、公交出行等便利服务，赢得了各方赞誉。

　　城市运行保障高效有力。实施全运场馆周边等重点区域环境综合整治，开通公交专线、循环班线，组织各类车辆3.3万台次，全力做好水、电、气、通信等各项公共服务，为赛事成功举办提供了重要保障。

　　出色完成安保工作。强化治安综合治理，严查严管食品药品安全，采取前端安检、驻地安保等有力措施，有效保证了各项赛事和活动的顺利进行，实现了“平安全运”目标。

　　三年来，全市人民齐心协力，参与全运、奉献全运，洒下了辛勤汗水。全运会的成功举办，为我们这座城市赢得了荣誉，留下了宝贵的精神财富，值得我们倍加珍惜。

　　（五）加大民生工作力度，社会更加稳定和谐

　　着眼于解决关系人民群众切身利益的实际问题，进一步加大民生投入，11件35项民生实事全部完成。

　　民生工作扎实推进。出台全民创业援助行动三年计划实施方案，困难家庭高校毕业生全部就业，零就业家庭保持动态为零，全市实名制就业21万人。城镇职工、城乡居民养老和医疗保险覆盖面不断扩大，企业退休人员基本养老金标准进一步提高，城镇居民医保和新农合年人均补助标准由240元提高到280元。提高最低工资、失业保险金、城乡低保标准，启动新农合大病保险，取消医疗救助最大封顶限额和定点医院限制。新增100个社区日间照料站，新增养老床位7000张，市养老服务中心二期主体完工。完成100个社区和4707户残疾人家庭无障碍改造，沈阳盲文图书馆和无障碍影院投入使用。完成保障性住房3.89万套，改造农村危房3608户，实施节能暖房工程408万平方米，新建改造76个菜市场，解决了15万农村人口饮水安全问题，减少农村低收入贫困人口2万余人。

　　社会事业全面发展。出台促进现代职业教育发展若干意见，调整完善中考制度，建立学前教育奖补机制，改扩建50所幼儿园，改造建设100所学校操场和14所特殊教育学校，和平区、铁西区成为国家级义务教育均衡发展示范区。医药卫生体制改革取得新进展，完成新民县级公立医院改革，启动康平县级公立医院改革，基层医疗卫生服务机构全部配备使用国家基本药物，并实行零差率销售，看病难、看病贵问题逐步得到缓解。繁荣艺术精品创作，发展多元化演出市场，实施艺术惠民工程，新编历史京剧《将军道》获年度国家舞台艺术十大精品工程奖，沈阳评剧院被评为全国地方戏创作演出重点院团，社区文化建设“五个一”工程运作模式成为国家公共文化服务体系示范项目，中国工业博物馆全部开放。建成一批市民休闲健身场所，开放104所中小学体育设施，全民健身掀起新热潮。

　　社会管理不断加强。出台加强社区建设若干意见，改善城乡社区服务设施，圆满完成第十一届村委会换届。加强社会治安立体防控，严厉打击违法犯罪，稳步推进“平安沈阳”建设。健全社会稳定风险评估机制，深入开展矛盾纠纷排查化解，强化安全生产和食品药品安全监管，有效应对H7N9禽流感疫情。全面推进文明城市创建，城市文明程度和市民文明素质进一步提升。

　　过去一年，我们坚持依法行政，积极转变政府职能，深入开展党的群众路线教育实践活动，努力改进工作作风，政府自身建设取得新成效。国防动员、人防和民兵预备役建设进一步加强，双拥创建工作全面推进。民族团结进步事业不断发展，对口支援工作成效显著。国家安全、保密、统计、审计、物价、工商行政管理、参事、宗教、侨务、贸促、广播电视、新闻出版、气象、防震减灾、档案、修志、文史、人口与计划生育、红十字会和慈善事业等工作都取得了新成绩。

　　各位代表，过去一年所取得的成绩来之不易，这是市委正确领导的结果，是全市人民团结一心、努力拼搏的结果。在此，我代表市政府，向全市各族人民致以崇高的敬意！向人大代表、政协委员，向各民主党派、工商联、无党派人士和各人民团体，向驻沈人民解放军、武警部队官兵和中、省直单位，向所有关心、支持沈阳的港澳台同胞、海外侨胞和国际友人表示衷心感谢！

　　在总结成绩的同时，我们也清醒地看到，全市经济社会发展还面临着一些突出的矛盾和问题。一是产业结构调整力度还不够大，经济运行质量和效益有待进一步提升；二是国家中心城市建设步伐还不够快，城市建设管理精细化水平需要进一步提高；三是一些关系人民群众切身利益的问题还没有解决到位，保障改善民生任务依然繁重。对上述问题，我们将采取有力措施认真加以解决。

　　二、2014年工作安排

　　今年是深入贯彻落实十八届三中全会精神的第一年，是确保完成“十二五”规划的关键之年，做好今年各项工作意义重大。今年政府工作的总体要求是：认真贯彻落实党中央、国务院和省委、省政府的决策部署，按照市委十二届八次全会要求，紧紧围绕“三大目标”、统筹推进“五大任务”，突出抓好深化改革、稳定增长、改善民生、优化环境等重点工作，加快转方式调结构步伐，努力提高经济增长质量和效益，促进经济平稳健康发展、社会和谐稳定。

　　今年全市经济社会发展的主要预期目标是：地区生产总值增长9%，固定资产投资增长15%，公共财政预算收入增长9.5%，社会消费品零售总额增长13%，城市居民人均可支配收入增长9%，农民人均纯收入增长10%，城镇登记失业率控制在4%以内。

　　今年，我们要着力抓好以下几方面工作。

　　（一）推进经济平稳健康发展

　　坚持稳中求进工作总基调，把稳定增长作为经济工作的首要任务，强化经济运行组织协调，努力保持经济增长的稳定性、协调性和可持续性。

　　加大项目建设力度。充分发挥投资的关键作用，突出抓好以290个市级重点项目为代表的一批重大产业项目。强化项目前期工作，做好土地、资金等要素保障，继续开展“双进双解”活动，及时提供各项服务，确保招商项目早日落地开工、在建项目早日竣工投产，尽快形成新的经济增长点。强化项目储备和跟踪落实，为未来发展积蓄后劲。

　　着力提升工业发展水平。瞄准建设具有国际竞争力的先进装备制造业基地，深入推进工业“五项工程”，实施创新驱动，深化两化融合，增强企业核心竞争力，规模以上工业增加值增长10%。加快装备制造业三大聚集区、两大配套区建设，提升汽车及零部件、机床及功能部件、电力电气、通用及专用机械、现代建筑、农产品加工、医药化工、手机等优势产业发展水平，积极创建国家现代建筑产业化示范城市。支持优势骨干企业做大做强，重视和关心中小微企业发展，进一步减轻企业负担，帮助企业解决多元融资、市场开拓、技术创新、人才引进等方面困难。实施战略性新兴产业发展三年行动计划，加快铁西、浑南、沈北三大新兴产业聚集区建设，培育壮大民用航空、IC装备、智能制造、电子信息、生物医药等新兴产业。加快重点园区和重大项目建设，大力发展工业地产，吸引配套企业加速集聚，进一步壮大产业集群。

　　大力发展服务业。围绕加快建设东北区域金融中心和物流、信息、科技创新中心，进一步完善政策体系，促进现代服务业特别是生产性服务业加速发展。支持沈阳金融商贸开发区、和平金融街和皇姑北站北等金融集聚区发展，大力引进各类金融机构，加快东北金融后援基地建设。加快沈阳国际物流港等7大物流集中发展区建设，提升物流业发展水平。推进浑南国家电子商务示范基地建设，鼓励支持企业发展电子商务，积极引进和设立区域性营运中心、结算中心，努力打造全国电子商务示范城市。积极引进大型商贸综合体项目，加快国际会展商贸城建设，办好制博会等品牌展会。推进国家级文化与科技融合示范基地建设，积极开发特色旅游资源，促进文化与科技、文化与旅游互动发展，提升城市文化软实力。继续保持房地产市场平稳健康发展。加快金廊建设步伐，努力打造高端中央商务区。推动太原街、中街等传统商业街区业态升级，大力发展夜间经济，积极培育健康养老、文化娱乐、信息服务等新的消费增长点。

　　加快发展农业和农村经济。落实强农惠农各项政策，促进农业发展、农民增收、农村和谐。加强农业基础设施建设，创建20万亩粮油高产综合示范区，保障粮食和主要农产品有效供给。大力发展县域经济，加快调整农业产业结构，开工建设80个规模以上农业产业化项目，支持辉山乳业集团建设世界级企业。抓好蒲河生态经济带、沈康现代农业示范带、环城都市农业产业带建设，发展现代都市农业和生态友好型农业，加快农业标准化、品牌化建设步伐。充分发挥比较优势，大力发展县域工业，不断壮大产业集群。推进“新网工程”建设，促进城乡市场有效衔接。加快县城和新城新市镇基础设施、市政配套设施和公共服务设施建设，增强城镇产业、人口聚集功能。

　　（二）提高城乡建设管理水平

　　坚持以完善城市功能为目标，以加强精细化管理为重点，进一步增强城市综合承载力，加快国家中心城市建设步伐。

　　加大基础设施建设力度。启动二环快速路二期、迎宾路高架桥、怒江北街三环公铁桥、云龙湖跨浑河桥、陵园街三环连接线建设和南北二干线快速路建设前期工作，续建谟家堡跨三环桥、东望街延长线、昆山东路延长线，加快地铁9号线、10号线和2号线北延线建设，推进地铁4号线和沈阳至新民、沈阳至辽中城际铁路建设前期工作。推进沈彰连接线改扩建、沈铁2号公路建设，启动京沈客专铁路建设。开工建设辽中绕城环线工程。实施沈阳北站北广场改造，加快新沈阳南站枢纽建设。实施浑河城市段南北两岸堤防工程，完善城市排水防涝体系，建设改造20个泵站，改造市区60处积水点，启动方家栏地区雨污分流工程。加快输水管道、配水管网等工程建设，实现大伙房水库输水工程一期90万吨供水目标，启动131万吨供水工程。推进大型热源建设和供热管网互联工程，加强地下管网建设、改造与管理。

　　继续改善生态环境。深入实施“蓝天”、“碧水”、“青山”工程，加强生态建设和环境治理。实施区域一体高效供热、气化沈阳、工业企业治理、城市抑尘、绿色交通、管理减排增效、大气监控预警等7项工程，积极采取措施，强化雾霾天气应对。巩固提升辽河、浑河、蒲河生态化治理成果，实施棋盘山秀湖综合环境整治，加强康平卧龙湖生态保护利用，启动细河、满堂河截污工程。推进垃圾分类、无害化处理、回收再利用，实施西部25万吨/日污水处理、祝家1000吨/日污泥处置、老虎冲垃圾渗沥液处理等项目，启动垃圾焚烧发电工程。完成三、四环沿线生态绿带和城区100条主要街路行道树补植。加大农村环境综合治理力度，完成农村环境连片整治示范工程。提高市、县（市）两级财政投入比重，确保“一市三县”垃圾收集处理系统稳定运行。完成全年节能减排任务。

　　提升城市精细化管理水平。进一步完善数字化城管系统，加大市政设施养护力度，打造50条高标准保洁精品街路。加快停车场、过街设施建设，继续更新公交车辆，积极创建“公交都市”。巩固提升机场、火车站、长途客运站、旅游风景区等窗口地区环境面貌，拆除违章建筑，规范广告牌匾，整治占道经营，开展历史文物挂牌及引导标识建设工作。继续推进文明城市创建，进一步提高城市文明程度和市民文明素质。

　　（三）扎实推进改革开放

　　坚持以改革增动力、增活力，以开放促改革、促发展，不断破除各方面体制机制弊端，进一步释放改革红利。

　　全面深化改革。深入推进新型工业化综合配套改革，提高改革的系统性、整体性和协同性，努力在行政体制、国有企业、非公经济、统筹城乡区域、金融体制、科技创新体制、民主政治制度建设、教育文化卫生事业、创新社会治理体制、生态文明体制等十个方面改革取得新突破。加快铁西区、东陵区（浑南新区）两化融合示范区建设，推进10大类公共服务平台、20户重点企业示范项目建设，着力打造国家级两化融合示范区。全面启动优化金融生态改革试验，深入开展金融创新，大力发展产业金融。推进大型国有企业完善现代企业制度，加快主辅分离，健全国有企业监管考评体系和激励约束机制，加快国有企业股权多元化改革，积极发展混合所有制企业。扩大市场准入，支持非公有制经济健康发展，不断激发市场活力。全面推进“低效用地”再开发，启动农村宅基地和集体建设用地使用权确权登记发证工作。发挥于洪统筹城乡发展、苏家屯新型城镇化、法库新型工业化和城镇化统筹发展等改革的示范作用，稳步推进新型城镇化。

　　着力增强自主创新能力。加快国家大学科技城建设，推进以企业为主体、以市场为导向、以高校院所为依托的产业技术创新战略联盟建设，鼓励自主创新，推进协同创新。加大科技研发投入，深入实施重大科技专项，进一步提升核心技术研发能力和科技成果转化能力。健全技术创新激励机制，促进科技成果资本化、产业化。加大领军人才和研发团队引进力度，为实现创新驱动发展和加快国家创新型城市建设提供有力支撑。

　　提高对外开放水平。加大对世界500强、行业领军企业和知名工业地产企业的招商力度，鼓励支持大企业（集团）在沈阳设立总部（区域总部）和研发中心，实现利用外资和吸引内资稳定增长。进一步提升沈阳综合保税区功能，加快出口基地建设。扶持本地企业做大做强，支持和帮助企业开拓海外市场、参与国际竞争。以沈抚、沈辽鞍海等城际连接带建设为重点，携手周边城市共同推动沈阳经济区一体化。

　　（四）着力保障改善民生

　　坚持以人为本，加大民生投入，继续为城乡群众办好一批实事，让全市人民共享振兴发展成果。

　　推动实现更高质量的就业。进一步完善就业创业扶持政策，拓宽高校毕业生创业就业渠道，全市实名制就业20万人。深入开展普惠制就业培训和农村转移劳动力就业培训，进一步提高复转军人创业能力，不断提高农民工就业能力。继续推进工资集体协商，推行集体合同制度，构建更加和谐的劳动关系。

　　完善多层次社会保障体系。进一步扩大社会保险覆盖面，提高城镇居民医保和新农合补助标准，启动城镇居民大病保险工作。提高城乡居民最低生活保障水平和助学、养老等专项救助标准。探索多渠道养老保障和服务方式，推动老龄事业健康发展。继续加大扶贫开发力度，大力发展社会福利和慈善事业。落实《沈阳市残疾人保障条例》，出台特殊贫困残疾人群体救助帮扶政策。

　　改善城乡居民生活条件。完成保障性住房2.37万套，改造21片零星棚户区和774户农村危房。实施650万平方米节能暖房工程，加强供热旧管网改造，提升供热服务水平。加强居民小区供水内网改造，继续实施法库新城及经济开发区给水配套工程，完成18.2万农村人口安全饮水改造任务。改善提升100个旧住宅小区环境质量，进一步提升旧住宅小区管理水平，完善新建住宅小区市政基础设施配套，新开调整10条公交线路，建设80处公交站务室、140处环卫工人休息室。继续整修小街小巷，改扩建500公里农村公路，新建150公里自然村之间柏油路，让城乡居民出行更便捷。

　　大力发展社会事业。全面启动教育现代化建设，继续实施百所幼儿园、百所义务教育学校提升工程，提高普通高中、职业教育发展水平，支持在沈高校发展。深化医药卫生体制改革，启动辽中、法库县级公立医院改革，加快卫生信息化建设，推进健康服务业发展。加快市一院二期、儿童医院二期、公共卫生服务中心等重点项目建设，不断提升专科医院诊疗水平。实施公共文化设施提升工程，改扩建市老年人大学和市少儿图书馆，新建市青少年活动中心和市妇女会馆，启动建设盛京博物馆，市艺术大厦、盛京文化艺术中心和宋雨桂艺术馆年内投入使用。完成万柳塘公园、八一公园、怒江公园景观提升及设施维修改造，开工建设舍利塔盛京碑林公园。建设15处市民休闲广场、18个乡镇文化站和117处农村社区文体广场，建成市全民健身室外运动广场，推动全运场馆加速向市民开放。

　　努力提高社会治理水平。改进社会治理方式，创新有效预防和化解社会矛盾体制机制，进一步健全公共安全体系。加大农村社区服务设施改造力度，加强城乡社区服务体系建设。提高企业安全生产标准化水平，推进安全生产网格化建设，强化电梯等公共设施规范管理和安全监察，有效防范重特大事故发生。着力构建长效监管机制，保障食品药品安全。健全社会治安防控体系，依法防范和惩治各类违法犯罪活动。加强诚信体系建设，营造良好发展环境。继续深化双拥共建，推进军民融合发展，加强国防后备力量建设，争创全国双拥模范城“八连冠”。

　　（五）加强政府自身建设

　　坚持以建设法治政府和服务型政府为目标，加快转变政府职能，加强公务员队伍建设，不断增强政府的公信力和执行力。

　　进一步提高依法行政水平。建立健全科学民主决策机制，完善重大公共决策群众参与制度，确保各项决策体现民意、符合实际。加强政府立法工作，强化行政执法监督，加大政务公开力度，自觉接受市人大及其常委会的依法监督和市政协的民主监督，坚持向人大报告工作和向政协通报工作，认真办理人大代表建议、议案和政协提案。

　　进一步转变政府职能。创新公共服务和社会管理体制机制，推动政府职能向创造良好发展环境、提供优质公共服务转变，进一步提高行政效能和服务水平。大力实施简政放权，优化行政审批流程，加强市、区县（市）、乡镇三级服务体系建设。健全有利于科学发展的考评机制和绩效管理体系，建立规范合理的政府债务管理和风险预警机制，更加注重发展质量、发展方式和发展后劲，不断提高推动科学发展的能力和水平。

　　进一步转变工作作风。政府各级领导干部要带头反“四风”、转作风，进一步增强服务意识，恪守为民之责，坚持求真务实，把精力用在干事业上。要直面矛盾，勇于担当，顾全大局，真抓实干，以奋发有为、昂扬向上的精神状态，努力开创各项工作的新局面，做出经得起实践、人民和历史检验的新业绩。

　　进一步加强廉政建设。建立健全惩治和预防腐败体系，增强制度约束力。严格遵守各项规定，厉行节约、反对浪费，严格控制三公经费支出。强化行政权力运行监督，严厉查处违法违纪案件。政府各级领导干部和全体公务员要进一步增强廉洁自律意识，提高拒腐防变能力，克己奉公，勤勉尽责，努力做人民满意的公仆。

　　各位代表，2014年是充满机遇和挑战的一年。让我们在市委的正确领导下，在市人大及其常委会依法监督和市政协民主监督下，锐意进取，埋头苦干，努力把沈阳老工业基地振兴发展推向新阶段！
